# Moving from isolation to integration: a Bayesian multisensory perspective of interoception and psychosis

**DOI:** 10.1038/s41386-025-02250-9

**Published:** 2025-09-24

**Authors:** Emma N. Herms, Krista M. Wisner

**Affiliations:** 1 Department of Psychological & Brain Sciences, Indiana University, Bloomington, IN USA; 2 Program in Neuroscience, Indiana University, Bloomington, IN USA

**Keywords:** Psychosis, Human behaviour, Decision

## Introduction

Interoception (sensing, integrating, and interpreting internal body signals [1]) has recently received increased attention, in part because of its critical contribution to affect, sense of self, and perception of the world [2]. However, interoceptive signals are never experienced in isolation; they are continuously influenced by, and in turn influence, other sensory inputs [3]. While this notion is widely accepted, many of the methods used to study interoception rely on uni-sensory paradigms that fail to reflect everyday multisensory experiences. By leveraging a Bayesian multisensory framework, we not only improve ecological validity (multisensory) but also quantify the contribution of individual sensory signals (Bayesian), to refine understanding of interoception in both everyday life and clinical experiences, such as psychosis-spectrum symptoms.

## Defining multisensory processing and Bayesian models

Multisensory processing refers to the interaction of multiple sensory signals, including signals about the body’s internal state (interoception), the body’s position and movement (proprioception), and the external world (exteroception), for a unified perceptual experience and adaptive behavior [4, 5]. For instance, something as simple as sipping hot coffee involves thermal feedback in the digestive tract, motor control, as well as visual and olfactory sensations.

**Fig. 1:**
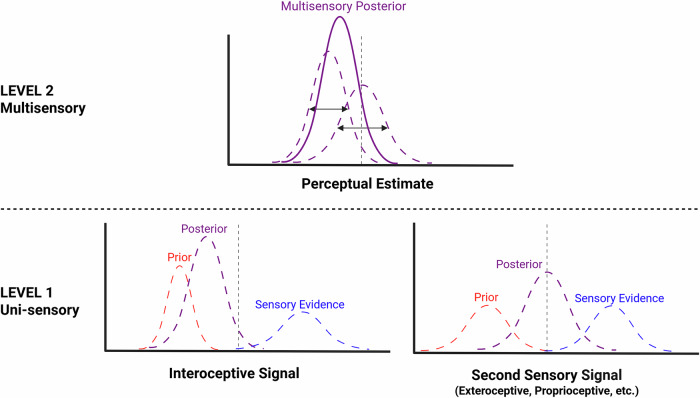
Illustrative representation of Bayesian precision-weighted integration of interoception with a second sensory signal. At Level 1 (bottom), each sensory signal is represented by priors (dashed red) and sensory evidence (dashed blue) that are combined based on their precision to form a signal-specific posterior (dashed purple). In this example, the interoceptive prior (left) is depicted as more precise and therefore exerts greater influence on the posterior distribution, whereas for the second sensory signal (right) the prior and sensory evidence are shown with equal precision and influence on the posterior. At Level 2 (top), these uni-sensory posteriors (dashed purple) are integrated into a single multisensory perceptual estimate (solid purple), again weighted by each signal’s precision (black arrows). Narrower distributions reflect higher precision, which exert greater influence on the multisensory posterior. This figure is conceptual and does not depict empirical data.

Multisensory processing can be modeled using a Bayesian framework. As depicted in Fig. 1, Level 1, priors (top-down influences) are combined with incoming sensory evidence (bottom-up signals) [6]. When applied to multisensory processing, this model predicts that different sensory modalities are weighted by their respective precision (Level 2) - a model-derived estimate of a signal’s reliability based on prior expectations and sensory evidence. For example, Ernst and Banks [7] demonstrated that when estimating the height of an object, people integrate visual and haptic information using a weighted average, with more ‘precise’ signals exerting greater influence on perception. Thus, Bayesian modeling provides additional value, on top of multisensory paradigms, by quantifying the precision-weighted contribution of a given sensory signal.

## A Bayesian multisensory lens of interoception in everyday life

Extending this framework to interoception can establish important principles and address a foundational challenge in interoception research: how precise are internal signals? Interoception often operates below conscious awareness unless homeostasis is disrupted [8]. Even when the magnitude of interoceptive signals is elevated, they tend to be spatially and temporally diffuse [9], potentially limiting their sensory precision. Thus, we hypothesize (1) interoceptive signals may exert less precision-weighted influence on perception relative to other sensory inputs, and (2) particularly at rest, interoceptive perception may be more strongly shaped by prior expectations than sensory evidence.

A Bayesian multisensory framework makes it possible to test these hypothesized principles in each interoceptive domain (e.g., cardiac, respiratory, temperature). For example, by incorporating controlled thermal stimuli into an Ernst and Banks-style paradigm [7], researchers could manipulate interoceptive and exteroceptive precision (e.g., varying signal noise) to capture their impact on multisensory judgments. This approach begins to address hypothesis 1, by quantifying interoception’s dynamic contribution to perception. Taking a different approach, Allen and colleagues demonstrated that simulated agents with reduced cardiac-interoceptive precision relied more heavily on interoceptive priors, which distorted their exteroceptive perception [10]. This computational proof-of-concept provides a basis for future experimental work to investigate when interoceptive perception, and perception more broadly, is shaped by interoceptive priors, towards addressing hypothesis 2.

Taken together, combining Bayesian models with multisensory paradigms allows researchers to quantify the precision-weighted contribution of interoception, relative to other sensory signals, and test key hypothesized principles of interoceptive functioning in everyday life. Establishing this foundation in the general population is essential for identifying how the relative weighting of interoceptive signals may differ in clinical groups or relate to specific symptoms.

## Interoception’s multisensory contribution to clinical symptoms: Psychosis as an exemplar

Interoception’s clinical relevance may not lie in the fidelity of any one internal body signal, but in how interoceptive signals are dynamically weighted and combined with other signals to shape experience. We illustrate this point using two psychosis-spectrum symptoms, disturbances in sense of self and hallucinations.

Disturbances in ‘sense of self’ (e.g., feeling fragmented, disembodied, lacking agency) are common in psychosis-spectrum disorders [11], but it remains unclear whether, and in what ways, altered weighting or integration of interoceptive signals contribute [12]. One way to capture these experiences is through body ownership paradigms like the rubber hand illusion. In recent interoceptive adaptations, affective touch (i.e., slow, caress-like touch) enhanced the illusion, highlighting how interoceptive signals can strengthen ownership of the rubber hand [13]. While not yet examined in psychosis samples, if affective touch signals fail to enhance the illusion, this indicates reduced precision-weighting of interoceptive information at the uni-sensory (Fig. 1, Level 1) or multisensory integration (Level 2) stage. Conversely, if affective touch signals enhance the illusion more than in comparison participants, this may reflect the overweighting of interoceptive signals at either level. Incorporating Bayesian modeling can disentangle these possibilities, providing mechanistic insight into symptom expression.

Bayesian theories and models have shown promise for reinterpreting quintessential psychosis symptoms like hallucinations. For example, Powers and colleagues found that clinical and non-clinical hallucinators had overly precise auditory priors, leading to perception of auditory stimuli when none were administered [14]. We are currently extending this approach to uni-sensory interoceptive thermal stimuli to test whether hallucinations are similarly associated with overly precise interoceptive priors (F31 MH136742-01). However, incorporating multisensory conditions is crucial for clarifying whether hallucinations reflect a general tendency toward overly precise priors across uni-sensory signals (Fig. 1, Level 1), or if they emerge through altered integration of signals (Level 2). It is possible that overly precise priors observed in exteroceptive domains (e.g., auditory) may in part reflect biasing from unmeasured interoceptive signals, a key source of affective salience that could increase vulnerability to hallucination experiences [15].

## Conclusion

In defining and measuring interoception, we often aim, in vain, to separate it from exteroception and proprioception. But this distinction is inherently circular. Interoception is always embedded in a multisensory context, shaped by and shaping other sensory inputs. In this commentary, we aimed to inspire research that embraces the complexity of our multisensory world. A Bayesian multisensory framework is crucial for establishing foundational principles of interoception in everyday life and improving conceptualization of their contributions to clinical symptoms.
